# Activity of aztreonam-avibactam, cefiderocol, and cefepime-taniborbactam against a global collection of genetically characterized metallo-β-lactamase-producing Enterobacterales

**DOI:** 10.1128/aac.00842-25

**Published:** 2025-12-05

**Authors:** Mariana Castanheira, John H. Kimbrough, Gina M. Morgan, Maura Karr, Helio S. Sader

**Affiliations:** 1 Element Materials Technology (JMI Laboratories) 138461 , North Liberty, Iowa, USA; Shionogi Inc., Florham Park, New Jersey, USA

**Keywords:** metallo-β-lactamases, Enterobacterales, new antimicrobial agents, susceptibility, resistance mechanisms

## Abstract

We evaluated the activity of aztreonam-avibactam and comparators tested against 490 MBL-producing Enterobacterales isolates collected in 27 countries during 2019–2022. The most common MBL was NDM-1, which was detected among 307 isolates. The genes encoding NDM-5 and VIM-1 were detected among 68 and 53 isolates, respectively. Other 14 MBL-encoding genes or combinations were detected among 62 isolates. Mexico, Turkey, and Greece had the highest number of isolates (60, 59, and 57, respectively). All isolates were susceptible to aztreonam-avibactam (MIC_50/90_, 0.12/0.5 mg/L) when applying the EUCAST or FDA recently approved breakpoints (≤4 mg/L susceptible). Cefiderocol inhibited 66.7%/90.8% of the isolates when using EUCAST/FDA breakpoints. Cefepime-taniborbactam was active against 36.7%/55.5% of the isolates using the cefepime breakpoint for comparison purposes. Tigecycline and colistin inhibited 94.1% and 76.6% of the isolates (FDA and EUCAST breakpoints, respectively). The analysis of resistance mechanisms and genetic background of the MBL-producing isolates demonstrated genetic diversity among main species and presence of multiple resistance mechanisms including other β-lactamases, porin changes, and disruptions of efflux pump repressors. Aztreonam-avibactam was active despite the presence of these additional resistance mechanisms, but cefiderocol and cefepime-taniborbactam displayed variable activity. PBP3 YRIN and YRIK insertions were observed among 30 MBL-producing *E. coli* isolates. Aztreonam-avibactam MIC values ranged from 0.03 to 4 mg/L for these isolates, including an isolate carrying *bla*_NDM-7_, *bla*_CMY-42_, and porin changes. MBL-producing organisms are still considered an unmet medical need. Aztreonam-avibactam was active against this large collection of MBL-producing isolates that had elevated MIC values for many comparator agents.

## INTRODUCTION

Acquired metallo-β-lactamases (MBLs) emerged in the early 1990s with the description of IMP-1 in a *Serratia marcescens* isolate from Japan (1). The description of this enzyme was followed by the report of a *Pseudomonas aeruginosa* isolate from Verona, Italy, that produced an MBL enzyme named VIM-1 (2). Despite the many reports of MBL-producing isolates in the literature subsequent to the description of IMP- and VIM-producing isolates, their prevalence was low and somehow limited to certain geographic areas until the late 2000s (3, 4). This scenario changed after reports of NDM-1 in 2009 (5). This MBL was initially described in India, but a decade after its discovery, NDM-1 became the most common MBL worldwide (6). Isolates producing NDM have been reported on all continents, and this gene has been reported among various Gram-negative species, but mainly among Enterobacterales species (7).

Different from most other β-lactamases belonging to Ambler classes A, C, and D that coordinate the cleavage of the β-lactam ring through its serine residues, class B MBLs require the presence of divalent cations to hydrolyze the β-lactam ring (8). Additionally, these enzymes do not have activity against monobactams, such as aztreonam (9). Despite this characteristic, Enterobacterales and other Gram-negative species carrying MBLs can be resistant to aztreonam not only due to the presence of other acquired β-lactamases or the overexpression of intrinsic β-lactamases but also penicillin binding protein (PBP) alterations and permeability changes (3).

Current clinically approved β-lactamase inhibitors do not have activity against MBL enzymes, and isolates producing MBLs are often multidrug-resistant (10). Thus, therapeutic options to treat these isolates are limited. In 2024, the European Medicines Agency (EMA) approved aztreonam-avibactam for the treatment of complicated Enterobacterales infections where treatment options are limited (11). More recently, aztreonam-avibactam was approved by the US Food and Drug Administration (FDA) for the treatment of complicated intra-abdominal infections (cIAI) in combination with metronidazole in adult patients with limited therapeutic options.

Aztreonam-avibactam has demonstrated *in vitro* activity against MBL-producing Enterobacterales isolates (12–14), since aztreonam is not hydrolyzed by the MBL, and avibactam, as a potent inhibitor of class A, C, and most D enzymes, protects aztreonam from the hydrolysis of other β-lactamases.

In this study, we expand on the knowledge of the *in vitro* activity of aztreonam-avibactam against a large collection of MBL-producing Enterobacterales isolates collected in hospitals located in the Asia-Pacific, Europe, and Latin America and compare its activity against that of cefiderocol, cefepime-taniborbactam, and other comparator agents. Additionally, we performed a comprehensive evaluation of other β-lactam resistance mechanisms among the main bacterial species carrying MBL-encoding genes.

## MATERIALS AND METHODS

### Bacterial isolates

A total of 33,366 Enterobacterales isolates were collected during 2019–2022 in hospitals located in Asia-Pacific (17 hospitals), Europe (44), and Latin America (11). Bacterial isolates determined to be the cause of infection by local clinical and/or microbiological criteria and only one isolate per patient episode were included in this investigation. Carbapenem-resistant Enterobacterales (CRE, using CLSI criteria) isolates were screened for the presence of β-lactamases, and the isolates producing MBLs were included in this study. Species identification was confirmed by MALDI-TOF MS using the Bruker Daltonics MALDI Biotyper (Billerica, MA) following the manufacturer’s instructions.

### Antimicrobial susceptibility testing

Antimicrobial susceptibility testing was performed by reference broth microdilution methods conducted according to CLSI procedures (15) using BD BBL cation adjusted Mueller-Hinton broth (Franklin Lakes, NJ). Cefiderocol was tested in iron-depleted Mueller-Hinton broth. Avibactam was tested at a fixed 4 mg/L concentration when combined to aztreonam or ceftazidime, and taniborbactam was tested at a fixed 4 mg/L when combined with cefepime. Antimicrobial agents were procured from Pfizer (avibactam; New York, NY), MedChem Express (cefiderocol and taniborbactam; Monmouth Junction, NJ), USP Pharmacopeia (Rockville, MD) or Sigma-Aldrich (St. Louis, MI).

Quality control (QC) was performed to ensure proper test conditions by concomitantly testing the following QC strains: *Escherichia coli* ATCC 25922 and NCTC 13353, *Klebsiella pneumoniae* ATCC 700603 and ATCC BAA-1705, and *Pseudomonas aeruginosa* ATCC 27853. EUCAST and US FDA clinical breakpoints were used for the interpretation of susceptibility rates (16). Cefepime alone susceptible breakpoint was applied to cefepime-taniborbactam.

### Characterization of β-lactam resistance mechanisms

All isolates displaying imipenem and/or meropenem MIC values ≥2 mg/L (CRE) were screened for the presence of β-lactamases by whole genome sequencing and data analysis. DNA samples were prepared for sequencing using the Nextera XT library construction protocol and index kit (Illumina, San Diego, California, USA) following the manufacturer’s instructions and then were sequenced on a MiSeq or NextSeq Sequencers (Illumina) with a target coverage of 30×. FASTQ format files for each sample set were assembled independently. An in-house-designed software using the target assembled sequences (17) as queries to align against numerous resistance determinants from the NCBI Bacterial Antimicrobial Resistance Reference Gene Database (https://www.ncbi.nlm.nih.gov/bioproject/PRJNA313047) was used to search for β-lactamase genes. Potential matches for β-lactamase and selected genes were generated with the criteria of >94% identity and 40% minimum coverage length when compared with reference sequences.

## RESULTS

Among 1,412 CRE isolates (4.2% of 33,366 total Enterobacterales isolates), MBL-encoding genes were detected among 490 isolates corresponding to 34.7% of the CRE isolates and 1.5% of the overall Enterobacterales isolates. *K. pneumoniae* was the most common species carrying MBL genes and accounted for 67.1% (329/490) of the isolates. Among the remaining MBL producers, 62 were *Enterobacter cloacae* species complex (*E. cloacae* herein), 43 *E. coli*, and 22 *Klebsiella oxytoca* and seven other species with less than 10 isolates: *Citrobacter amalonaticus/farmeri* (1), *Citrobacter freundii* species complex (9), *Klebsiella aerogenes* (1), *Proteus mirabilis* (5), *Providencia rettgeri* (3), *Providencia stuartii* (8), and *Serratia marcescens* (7). MBL-producing isolates were recovered mainly from patients hospitalized with pneumonia (*n* = 146), bacteremia (*n* = 112), and urinary tract infections (*n* = 102) but also skin and soft tissue infections (*n* = 79), intra-abdominal infections (*n* = 41), and other or unknown infection sites (*n* = 10).

The most common MBL was NDM-1, which was detected among 307 isolates alone or in combination with other MBL genes in three additional isolates (Table 1). The genes encoding NDM-5 and VIM-1 were detected among 68 and 53 isolates, respectively. Eleven other MBL variants were detected among 59 isolates.

**TABLE 1:** Distribution of MBL-producing Enterobacterales isolates by continent and country^*a*^

Continent/ Country	No. of isolates							
	All MBLs (*n* = 490)	NDM-1 (*n* = 307)	NDM-4 (*n* = 13)	NDM-5 (*n* = 68)	NDM-7 (n = 25)	IMP-8 (*n* = 8)	VIM-1 (*n* = 53)	Other MBLs (*n* = 16)
Asia-Pacific	135	57	13	37	18	5	3	3
Australia	3			2				IMP-4 (1)
Korea	5	2		3				
Malaysia	45	19	10	15				NDM-1, NDM-5 (1)
Philippines	32	8		6	18			
Taiwan	12	4				5	3	
Thailand	27	18	1	8				
Turkey	59	56		2				IMP-1 (1)
Vietnam	11	6	2	3				
Europe	252	175		15	4		46	11
Belarus	18	17		1				
Belgium	2			2				
France	16	6		6	3			NDM-19 (1)
Germany	8	5					2	VIM-23 (1)
Greece	57	31					23	VIM-19 (3)
Ireland	2							NDM-9 (2)
Israel	4	2		1			1	
Italy	23	9		2			12	
Poland	23	19					1	NDM-1+VIM-1 (1), NDM-6 (1), VIM-4 (1)
Romania	19	18						VIM-4 (1)
Russia	11	10		1				
Slovenia	1	1						
Spain	8	1					7	
United Kingdom	1				1			
Latin America	103	75		16	3	3	4	2
Argentina	12	4		5		3		
Brazil	12	12						
Chile	7				3		4	
Mexico	60	53		5				NDM-1+NDM-5 (1), VIM-23 (1)
Panama	12	6		6				

^*a*^ The overall by continent is highlighted in gray.

MBL-producing Enterobacterales were recovered in 27 countries: 252 isolates detected in European countries, 135 in the Asia-Pacific, and 103 in Latin America (Table 1). The countries with the highest numbers of MBL isolates were Mexico, Turkey, and Greece (60, 59, and 57 isolates, respectively).

Aztreonam-avibactam (MIC_50/90_, 0.12/0.5 mg/L) inhibited all 490 isolates at ≤4 mg/L, the EUCAST/US FDA recently approved susceptibility breakpoints for this agent (Table 2). Cefiderocol (MIC_50/90_, 2/4 mg/L) inhibited 66.7% of the MBL-producing isolates using the EUCAST breakpoint, but 90.8% of the isolates were inhibited by this agent when applying the US FDA breakpoints. Cefepime-taniborbactam (MIC_50/90_, 2/32 mg/L) inhibited 36.7% and 55.5% of the isolates when applying the EUCAST and US FDA breakpoints for cefepime alone for comparison purposes, respectively. All other β-lactam agents inhibited <20% of the isolates tested. Among other antimicrobial classes, tigecycline (MIC_50/90_, 0.5/2 mg/L) inhibited 94.1% of the isolates applying the US FDA breakpoint, and 76.6% of the isolates were susceptible to colistin (MIC_50/90_, 0.25/>8 mg/L) using the EUCAST breakpoint. Gentamicin (MIC_50/90_, >16/>16 mg/L) was active against 34.1% of these isolates, while amikacin (MIC_50/90_, 16/>32 mg/L) inhibited 47.1% of the isolates using EUCAST breakpoints.

**TABLE 2:** Activity of aztreonam-avibactam and comparator agents against 490 MBL-producing Enterobacterales isolates

Antimicrobial agent	% of isolates inhibited at MIC (mg/L)						MIC_50_ (mg/L)	MIC_90_ (mg/L)	EUCAST %susceptible	US FDA %susceptible
	≤0.5	1	2	4	8	16				
Aztreonam-avibactam	93.9	96.9	99.2	100.0			0.12	0.5	100	100
Cefiderocol	12.9	32.4	66.7	90.8	96.3	99.0	2	4	66.7	90.8
Cefepime-taniborbactam	19.4	36.7	55.5	65.7	73.1	80.8	2	32	36.7^*a*^	55.5^*a*^
Ceftazidime-avibactam	1.8	1.8	1.8	2.0	2.2	2.7	>32	>32	2.2	2.2
Aztreonam	12.7	14.7	15.5	17.1	18.4	22.2	>16	>16	14.7	17.1
Cefepime	0.0	0.2	0.4	0.8	1.0	5.1	>32	>32	0.2	0.4
Piperacillin-tazobactam			0.0	0.2	1.0	1.2	>128	>128	1.0	1.0
Meropenem	1.0	2.4	6.3	12.4	20.8	33.3	32	>32	6.3	2.4
Levofloxacin	12.7	23.9	28.0	32.5	40.5	61.6	16	>32	12.7	12.7
Gentamicin	22.0	31.2	34.1	37.8	42.9	47.1	>16	>16	34.1	37.8
Amikacin	0.2	7.1	16.9	32.4	47.1	62.4	16	>32	47.1	62.4
Trimethoprim-sulfamethoxazole	9.4	13.7	15.7	17.1			>4	>4	15.7	15.7
Tigecycline	60.2	81.6	94.1	99.2	99.6		0.5	2	NA^*b*^	94.1
Colistin	75.2	75.8	76.6	77.0	79.5		0.25	>8	76.6	NA^*b*^

^*a*^ Cefepime-taniborbactam breakpoints have not been approved, and the susceptibility rate is based on the breakpoints for cefepime alone.

^*b*^ NA, not available.

**Fig 1:**
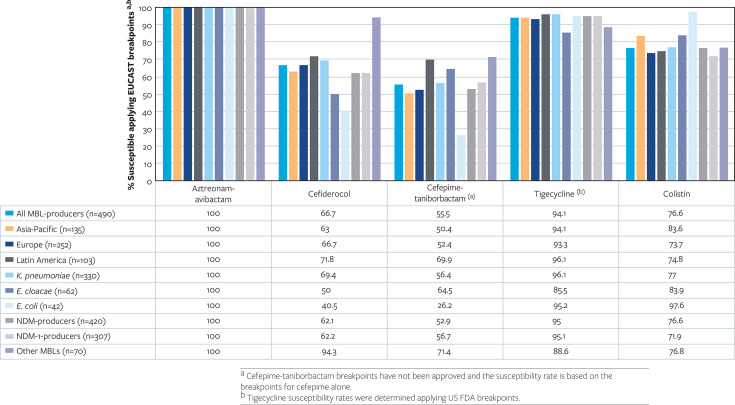
Activity of aztreonam-avibactam, cefiderocol, cefepime-taniborbactam, tigecycline, and colistin against MBL-producing Enterobacterales stratified by country, organism, and MBL type.

Aztreonam-avibactam was active regardless of the geography, organism, or MBL type (Fig. 1), but this was not observed for other comparators with *in vitro* activity against MBL-producing Enterobacterales. The activity of cefiderocol ranged from 63.0% to 71.8% (EUCAST breakpoints) in the different continents, while cefepime-taniborbactam, tigecycline, and colistin varied from 50.4% to 69.9%, 93.3% to 96.1%, and 73.7% to 83.6%, respectively (EUCAST breakpoints for all but tigecycline for which US FDA breakpoints were applied). Isolates from Latin America displayed the highest susceptibility rates for cefiderocol, cefepime-taniborbactam, and tigecycline. These comparators also displayed different activity against the main species carrying MBLs (Fig. 1). *K. pneumoniae* isolates displayed higher susceptibility rates for cefiderocol (69.3% susceptible; MIC_50/90_, 2/4 mg/L) and tigecycline (96.0%; MIC_50/90_, 0.5/2 mg/L) when compared with *E. cloacae* (50.0% and 85.5%, respectively) and *E. coli* (41.9%; MIC_50/90_, 4/16 mg/L and 95.2%; MIC_50/90_, 0.25/0.5 mg/L). Cefepime-taniborbactam exhibited limited activity against *E. coli* isolates producing MBLs, inhibiting only 27.9% of the isolates at the cefepime EUCAST susceptibility breakpoint while inhibiting 56.2% of the *K. pneumoniae* (MIC_50/90_, 2/32 mg/L) and 64.5% of the *E. cloacae* isolates. Finally, isolates carrying any NDM variant or NDM-1 enzymes exhibited lower susceptibility rates to cefiderocol (62.1% and 62.2%) and cefepime-taniborbactam (52.9% and 56.7%) than isolates producing other MBLs (94.3% for cefiderocol and 71.4% for cefepime-taniborbactam).

**Fig 2:**
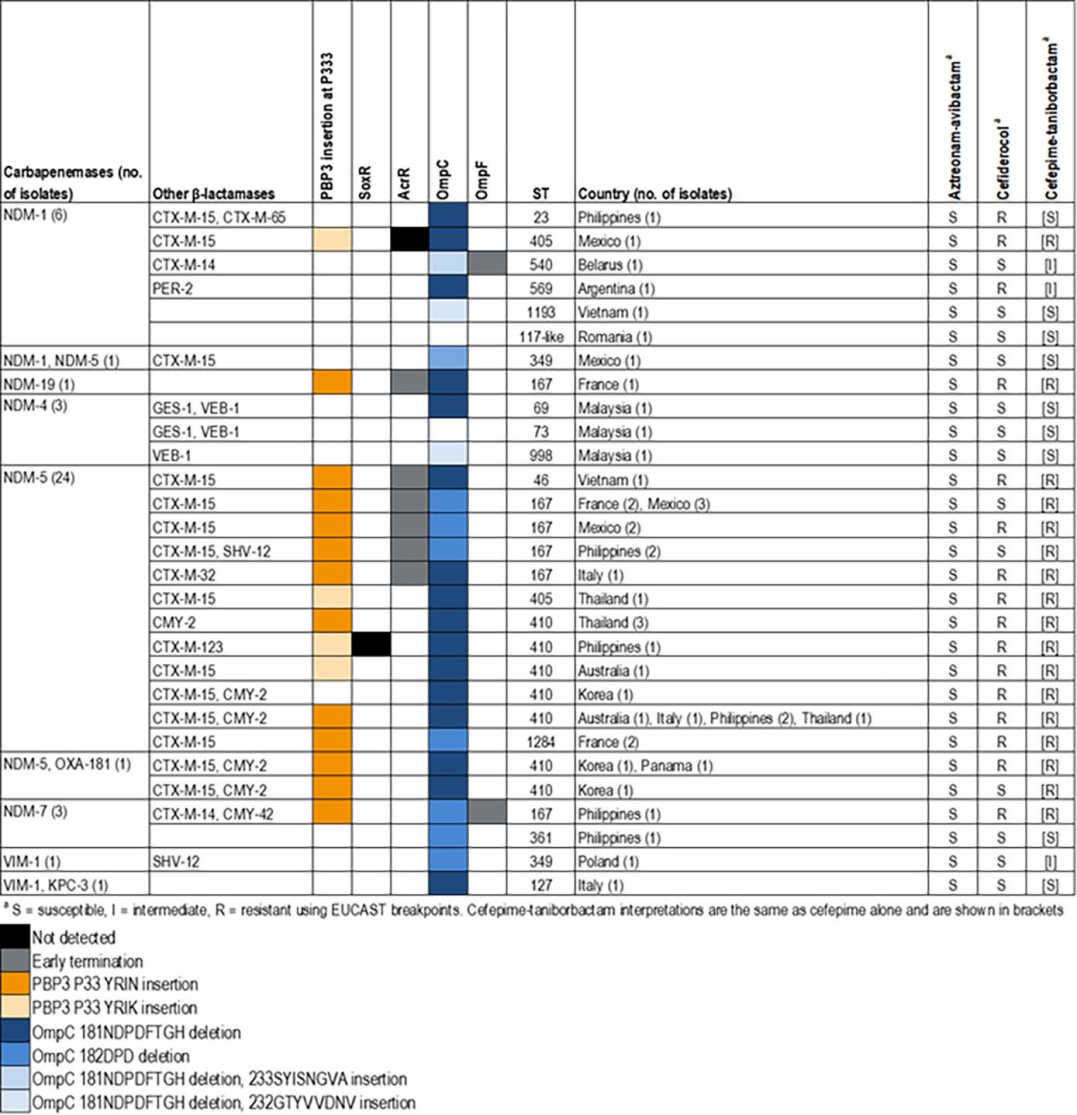
Genetic characteristics of MBL-producing *E. coli* isolates.

The genetic evaluation of the 43 MBL-producing *E. coli* isolates demonstrated that isolates belonged to important clones, including ST410 (14 isolates) and ST167 (12 isolates) that were detected in various countries (Fig. 2). Fourteen other sequence types (STs) were also observed. MBL-producing *E. coli* isolates carried additional β-lactam resistance mechanisms. Most (39/43) isolates carried ESBL- or CMY-encoding genes, most commonly *bla*_CTX-M-15_ with or without *bla*_CMY-2_. A total of 30 isolates carried PBP3 insertions YRIN (26 isolates) or YRIK (four isolates) at position 33 previously described to increase the MIC values for aztreonam-avibactam. These isolates displayed aztreonam-avibactam MIC values ranging from ≤0.03 to 4 mg/L (mode, 2 mg/L; 10 exhibited MIC values ≤0.5 mg/L) and were all categorized as susceptible when applying the recently approved breakpoint approved by EUCAST/US FDA for this combination. Among isolates carrying PBP3 insertions, one harbored *bla*_NDM-7_, *bla,*_CMY-42_ and disruptions and early termination of OmpC and OmpF, respectively. This isolate displayed an aztreonam-avibactam MIC value at 4 mg/L. Notably, all but one of the isolates harboring PBP3 YRIN or YRIK insertions were resistant to cefepime-taniborbactam, and 17/30 (56.6%) isolates were resistant to cefiderocol when applying the EUCAST breakpoint criteria.

All MBL-producing *E. coli* isolates belonging to ST167 and one isolate belonging to ST46 displayed early termination of AcrR that regulates the AcrAB-TolC efflux pump, and all but two isolates displayed deletions in the β8 sheet of OmpC, alone or with other alterations.

**Fig 3:**
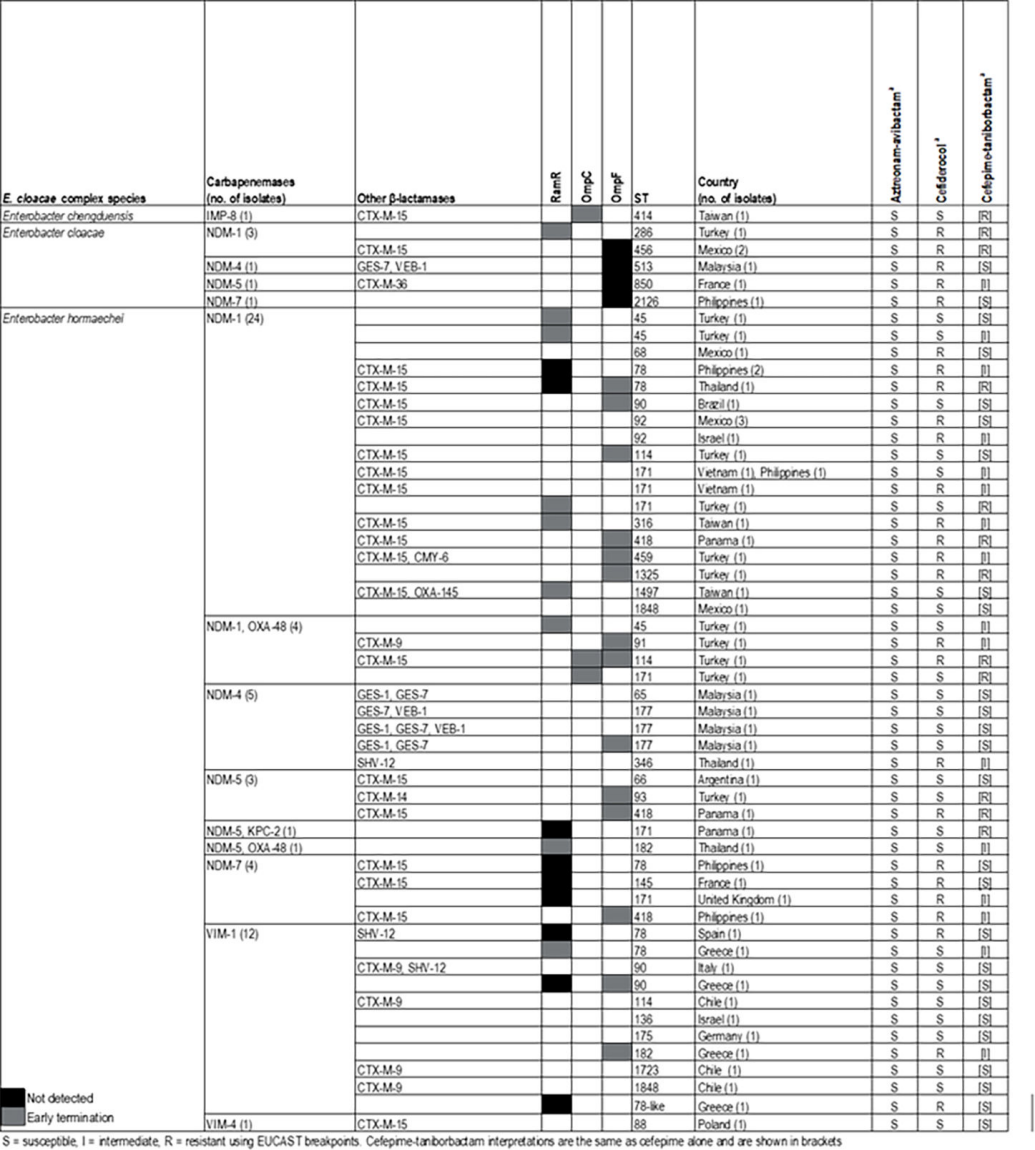
Genetic characteristics of MBL-producing *E. cloacae* species complex isolates.

MBL-producing *E. cloacae* species complex was mostly *Enterobacter hormaechei* (55/62) but also *E. cloacae* (six isolates) and one *E. chengduensis* carrying IMP-8 (Fig. 3). Overall, *E. hormaechei* and *E. cloacae* were genetically diverse with no ST having more than seven isolates (ST171 had seven isolates), and most isolates carried NDM-encoding genes (48/62). CTX-M, GES, and VEB variants and SHV-12 were observed in 41 isolates alone or in combination. Interestingly, all isolates from Malaysia carried GES and/or VEB enzymes regardless of species or genetic background. Early termination of OmpF was detected in 14 isolates without correlation with resistance to aztreonam-avibactam (all isolates were susceptible), cefiderocol, or cefepime-taniborbactam (Fig. 3). Absence or early termination of RamA (10 and 9 isolates), an efflux pump regulator, and the early termination of OmpC (three isolates) were also observed.

A total of 285 *K*. *pneumoniae* isolates (*n* = 329) producing MBLs co-harbored ESBLs and/or CMY enzymes. Similar to MBL-producing *E. coli* and *E. cloacae* isolates, *bla*_CTX-M-15_ was commonly observed among MBL-producing *K. pneumoniae* and was noted alone or in combination with other enzymes among 267 isolates. Beyond acquired β-lactamases, the efflux pump regulators AcrR and RamR had an early termination (6 and 29 isolates, respectively), major disruptions that likely impaired function (12 and 3) or were not detected (only RamR; 11 isolates). Additionally, early termination of OmpK35 and OmpK36 was noted among 90 and 7 isolates, respectively, with two additional isolates carrying no OmpK36-encoding gene. A total of 77 isolates harbored the insertion of a glycine, an aspartate, or both at position 132 (119 of the mature protein) that have been described to generate a pore constriction that doesn’t allow for the entrance of several β-lactams (18).

**Fig 4:**
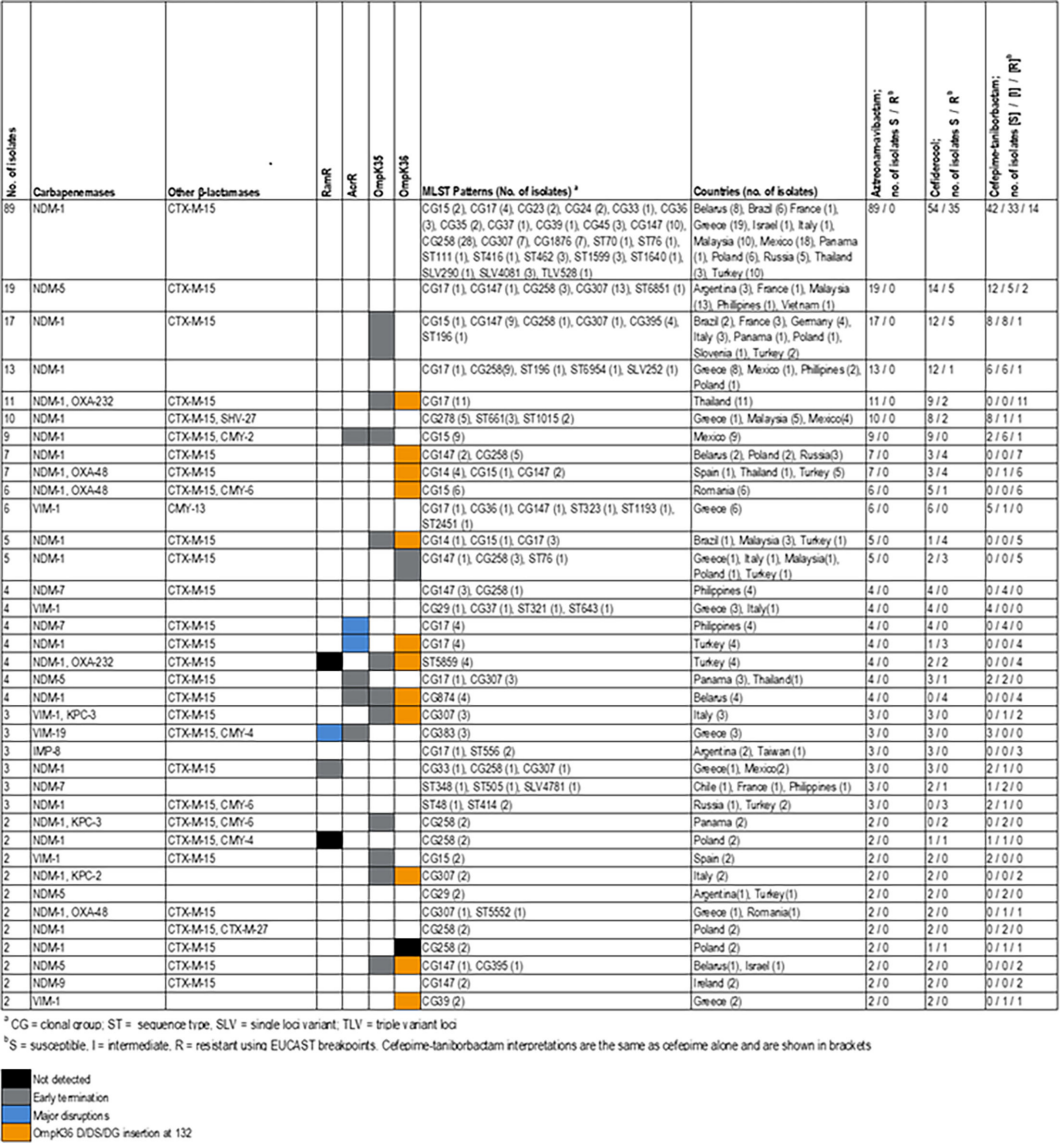
Genetic characteristics of 272/329 MBL-producing *K. pneumoniae* isolates with resistance mechanism patterns noted in ≥ 2 isolates.

MBL-producing *K. pneumoniae* belonged to at least 78 MLST profiles that included clonal groups (CGs), sequence types (STs), single loci variants (SLVs), and triple loci variants (TLVs), with 94 different combinations of resistance mechanisms. The combinations observed in ≥2 isolates are listed in Fig. 4, and isolates displaying single resistance mechanism combinations are listed in the Supplemental Table. A combination of *bla*_NDM-1_, and *bla*_CTX-M-15_ was detected among 89 isolates, 13 countries, and 24 genetic backgrounds (STs). All isolates displaying this enzyme combination were susceptible to aztreonam-avibactam, but 35 (39.3%) were resistant to cefiderocol, and 14 (16.5%) were resistant to cefepime-taniborbactam when applying the cefepime EUCAST breakpoints.

Regardless of the combination of resistance mechanisms, aztreonam-avibactam MIC values were susceptible when applying the EUCAST breakpoints (Fig. 4; Table S1); however, the susceptibility of cefiderocol and cefepime-taniborbactam varied among isolates with different resistance mechanisms. Cefepime-taniborbactam resistance was noted among all 62 isolates exhibiting 13 combinations of resistance mechanisms (Fig. 4). Those include isolates producing IMP-8 (three isolates) and NDM-9 (two isolates), enzymes that are not inhibited by taniborbactam (19, 20), and combinations of *bla*_NDM-1_, *bla,*_CTX-M-15_ and OmpK36 alterations with or without other resistance mechanisms.

## DISCUSSION

Carbapenem-resistant Enterobacterales (CRE) isolates have been identified as a threat to human health (21, 22). Therapies targeted to treat CRE isolates that produce serine-carbapenemases have been in clinical use for a few years, but options for the treatment of MBL-producing Enterobacterales isolates are limited and β-lactam/β-lactamase inhibitor combinations active against MBLs were under different stages of development (23). The need for therapies that are active against MBL-producing Enterobacterales isolates has been underlined by the worldwide spread of these isolates (24), recent reports of increase in MBL-producing isolates (14, 25, 26), and higher mortality associated with infections caused by MBL-producing isolates (27). A report of the SENTRY Antimicrobial Resistance Program documented the increase in MBL-producing isolates in US hospitals during 2022 (28). This increase was driven mainly due to the spread of NDM-producing isolates. In a recent review, Mojica et al. (7) compiled the emergence of the 29 NDM variants described since 2011 and their global distribution that reaches all continents. The authors also urge the development of MBL inhibitors that have activity against MBL-producing isolates since currently approved inhibitors have no activity against these enzymes.

Avibactam has been approved for clinical use combined with ceftazidime since 2015 (29). Pairing avibactam with aztreonam that is not hydrolyzed by MBLs is a strategy to allow this monobactam to evade other β-lactamases and inhibit cell wall. The combination aztreonam-avibactam was recently approved by the EMA and the US FDA (30).

In this study, we evaluated the activity of aztreonam-avibactam, cefepime-taniborbactam, cefiderocol, and comparator agents against a large collection of MBLs collected as part of the SENTRY Antimicrobial Surveillance Program in Asia-Pacific, Europe, and Latin America. Aztreonam-avibactam was active against all the tested isolates at ≤4 mg/L regardless of the MBL type, organism, and presence of other resistance mechanisms. These additional resistance mechanisms were evaluated among *E. coli*, *E. cloacae,* and *K. pneumoniae*. Expectedly, many isolates of these three important bacterial species carried multiple resistance mechanisms that included combinations of any of the following: serine-carbapenemases, ESBLs, transferable AmpCs, disruptions in outer membrane proteins and deletion, early termination, or major disruptions in efflux pumps repressors (AcrR, SoxR, and RamR). In specific organisms, PBP3 alterations YRIN and YRIK were noted among *E. coli* isolates producing mainly NDM-5, but also NDM-1, NDM-7, and NDM-19, and most of these isolates carried CTX-M-15 and/or CMY-2 with outer membrane protein disruptions and early termination of AcrR. The *E. coli* isolates harboring these traits displayed aztreonam-avibactam MIC values ranging from ≤0.03 to 4 mg/L, and the highest MIC was displayed in one isolate carrying genes encoding NDM-7, CMY-42, and CTX-M-14, and disruption in both OmpC and OmpF. *E. coli* isolates exhibiting elevated aztreonam-avibactam MIC values producing NDM enzymes, CMY-42, and displaying permeability alteration have been reported in the literature (31, 32), but as demonstrated in this study, they are not common, and the aztreonam-avibactam MIC values are still categorized as susceptible according to the EUCAST and FDA approved breakpoints. Notably, cefiderocol was only active against 8/30 of the NDM-producing *E. coli* isolates carrying PBP3 insertions, and all these isolates were resistant to cefepime-taniborbactam when applying the cefepime breakpoints.

PBP3 alterations were not observed among MBL-producing *K. pneumoniae* and *E. cloacae* species complex isolates, but both organisms displayed high genetic diversity and additional resistance mechanisms with variable MIC values for cefiderocol and cefepime-taniborbactam. Despite the presence of these other resistance mechanisms, aztreonam-avibactam was active against all isolates. Other Enterobacterales were not evaluated for the presence of additional resistance mechanisms due to the complexity of finding reference sequences for intrinsic genes involved in β-lactam resistance.

### Conclusions

Infections caused by CREs and MBL isolates have been demonstrated to have higher attributable mortality rates than those caused by carbapenem-susceptible organisms, with delayed appropriate antimicrobial therapy playing a significant role in the CRE mortality (33). Options for the treatment of serine-carbapenemase-producing isolates have been clinically available for many years, but the recent increase in MBL-producing isolates is a concern. Understanding what type of carbapenemase is present in the organism causing the infection can guide appropriate antimicrobial therapy. Aztreonam-avibactam was recently approved in Europe and the United States, and this study confirms the excellent activity of this combination against a large and diverse collection of MBL-producing isolates from Asia-Pacific, Europe, and Latin America. Cefiderocol was active against <70% of the isolates, and cefepime-taniborbactam, which is still under evaluation by the FDA, had limitations against IMP variants, NDM-9, and many isolates carrying multiple resistance mechanisms, including *E. coli* isolates displaying PBP3 alterations.

## Data Availability

The Sequences analyzed in this study were deposited under bio project number PRJNA1322463, and the SRA accession numbers SAMN51245563 to SAMN51246052.

## SUPPLEMENTAL MATERIAL

Table S1 aac.00842-25-s0001.docx Genetic characteristics of *K. pneumoniae* isolates.

ASM does not own the copyrights to Supplemental Material that may be linked to, or accessed through, an article. The authors have granted ASM a non-exclusive, world-wide license to publish the Supplemental Material files. Please contact the corresponding author directly for reuse.
