# Supplementary material for: Activity of aztreonam-avibactam, cefiderocol, and cefepime-taniborbactam against a global collection of genetically characterized metallo-β-lactamase-producing Enterobacterales
Source: Antimicrob Agents Chemother. 2025 Dec 5;70(1):e00842-25. doi: 10.1128/aac.00842-25 (PMC12777553; doi:10.1128/aac.00842-25)
Supplement: Table S1 — Genetic characteristics of K. pneumoniae isolates. [file aac.00842-25-s0001.docx]

**Supplemental Table** Genetic characteristics of 57/329 MBL-producing *K. pneumoniae* isolates with resistance mechanisms patterns noted in single isolates
